# Pleiotrophin (PTN) Expression and Function and in the Mouse Mammary Gland and Mammary Epithelial Cells

**DOI:** 10.1371/journal.pone.0047876

**Published:** 2012-10-15

**Authors:** Sonia M. Rosenfield, Emma T. Bowden, Shani Cohen-Missner, Krissa A. Gibby, Virginie Ory, Ralf T. Henke, Anna T. Riegel, Anton Wellstein

**Affiliations:** 1 Lombardi Cancer Center, Georgetown University, Washington, District of Columbia, United States of America; 2 MedImmune, Gaithersburg, Maryland, United States of America; Baylor College of Medicine, United States of America

## Abstract

Expression of the heparin-binding growth factor, pleiotrophin (PTN) in the mammary gland has been reported but its function during mammary gland development is not known. We examined the expression of PTN and its receptor ALK (Anaplastic Lymphoma Kinase) at various stages of mouse mammary gland development and found that their expression in epithelial cells is regulated in parallel during pregnancy. A 30-fold downregulation of PTN mRNA expression was observed during mid-pregnancy when the mammary gland undergoes lobular-alveolar differentiation. After weaning of pups, PTN expression was restored although baseline expression of PTN was reduced significantly in mammary glands of mice that had undergone multiple pregnancies. We found PTN expressed in epithelial cells of the mammary gland and thus used a monoclonal anti-PTN blocking antibody to elucidate its function in cultured mammary epithelial cells (MECs) as well as during gland development. Real-time impedance monitoring of MECs growth, migration and invasion during anti-PTN blocking antibody treatment showed that MECs motility and invasion but not proliferation depend on the activity of endogenous PTN. Increased number of mammospheres with laminin deposition after anti-PTN blocking antibody treatment of MECs in 3D culture and expression of progenitor markers suggest that the endogenously expressed PTN inhibits the expansion and differentiation of epithelial progenitor cells by disrupting cell-matrix adhesion. In *vivo*, PTN activity was found to inhibit ductal outgrowth and branching via the inhibition of phospho ERK1/2 signaling in the mammary epithelial cells. We conclude that PTN delays the maturation of the mammary gland by maintaining mammary epithelial cells in a progenitor phenotype and by inhibiting their differentiation during mammary gland development.

## Introduction

The heparin-binding growth factor pleiotrophin (PTN; also known as HB-GAM or HARP; reviewed in [1]) is a 15 kDa secreted protein named for its pleiotrophic effects on different cell types. In mice, PTN and its tyrosine kinase receptor ALK are prominently expressed during embryonic and early postnatal development of the central and peripheral nervous system [2], [3], [4], [5], [6]. In addition to neuronal functions, PTN contributes to the epithelial-mesenchymal interactions in organs undergoing branching morphogenesis and in bone formation [7], [8], [9], [10], [11], [12]. In the adult, PTN and ALK expressions are downregulated in most tissues, with residual expression in the nervous system [4], [13].

An increase in PTN expression has been observed in the uterus during gestation, in the human placenta and during bone repair, inflammatory diseases and in human cancers [2], [13], [14], [15], [16], [17], [18], [19]. The biological effects of PTN-mediated signaling include neurite outgrowth and repair, angiogenesis and mitogenesis of fibroblasts, endothelial and some tumor cell lines [19], [20], [21], [22], [23]. PTN signaling can induce the activation of both the MAPK and PI3 kinase pathways in epithelial cells [24] and a direct role for PTN in mitogenesis and anti-apoptotic signaling was previously reported [19], [25], [26], [27], [28]. PTN activity has recently been shown to stimulate the differentiation and expansion of neuronal stem cells, osteoprogenitors and bone marrow stem cells [29], [30], [31].

The PTN protein was first purified from the supernatants of human breast cancer cells [32]. PTN mRNA expression has been shown in different estrogen receptor negative breast cancer cell lines and in primary breast cancers derived from different patients [19]. In NIH3T3 fibroblasts, PTN leads to transformation, whereas overexpression of a dominant negative, mutant PTN protein reverses the transformed phenotype of breast and other cancer cell lines [26], [33]. PTN has also been shown to be an angiogenic factor during tumor growth and a promoter of invasion and metastasis in different tumor types including breast cancers [19], [22], [34], [35]. A role for PTN-mediated signaling through the receptor tyrosine kinase ALK as well as the receptor phosphatase PTPRz has been suggested in breast cancers [36], [37], [38].

In normal human breast tissues, PTN is expressed in the epithelial, myoepithelial and endothelial cells [39]. In the mouse mammary gland, PTN expression has been described in both epithelial cells and adipocytes [40], [41]. Marzan et al. showed that PTN expression in differentiated adipocytes of the mammary gland was regulated by the retinoid acid receptor (RAR) and that PTN activity in the adipocytes was involved in the migration of epithelial ductal cells [40]. PTN mRNA expression has also been reported in mammary epithelial ducts by in *situ* hybridization [41]. However, the function of PTN in mammary epithelial cells is still unexplored. Finally, no mammary gland phenotype was described in PTN knock-out mice though they have an increased hippocampal activity [5], [6]. Previous data suggest a temporal regulation of PTN expression during pregnancy and a permanent downregulation of this growth factor in the mammary gland induced by parity [42]. A protective effect of early parity from breast cancer has been suggested by epidemiological studies [43], [44] as well as from carcinogen-induced breast cancer models in rats [45], [46], [47]. A better understanding of PTN function and regulation during mammary gland development could help to understand the role of PTN during breast cancer development and progression.

Here we show that PTN expression is regulated in mouse mammary glands both temporally and spatially during pregnancy and is affected by multiparity. A 30-fold downregulation of PTN expression was observed during mid-pregnancy when the mammary epithelial cells (MECs) start undergoing lobular-alveolar differentiation. We also found that blocking PTN activity caused enhanced maturation of the mammary gland accompanied by activation of the ERK1/2 signaling pathway in the epithelial compartment of the mammary gland. We show that PTN activity sustains motility and invasion of MECs grown on plastic and that blocking PTN activity caused increased number of mammospheres due to a more polarized structural organization shown by laminin deposition and a more differentiated phenotype as indicated by the expression of progenitor cell markers CD29, CD49f, SCA-1 and CD10.

## Results

### Temporal and spatial expression of PTN in the mouse mammary gland during pregnancy

**Figure 1:**
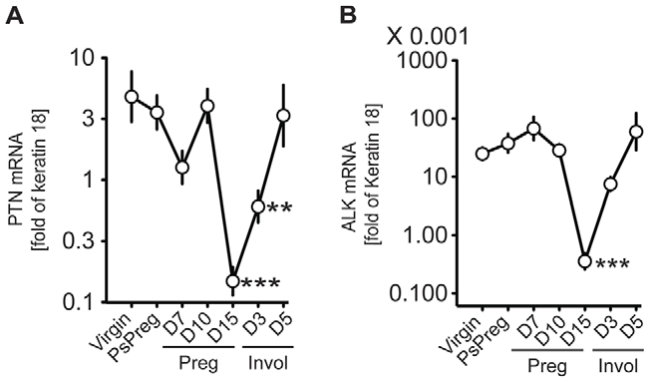
PTN and ALK mRNA expression in mouse mammary glands during pregnancy. Mammary glands from mice that were virgin, pseudopregnant (PsPreg), at different days of pregnancy or after weaning ( = involution). Quantitative, real-time PCR was used. Pseudopregnants denotes 12 days since plug date. Pups were removed from the mother at 21 days of age and involution followed thereafter. Expression is given relative to keratin 18. Mean ± SE (n = 4; PsPreg n = 2). **P<0.01; ***P<0.001, by ANOVA.

**Figure 2:**
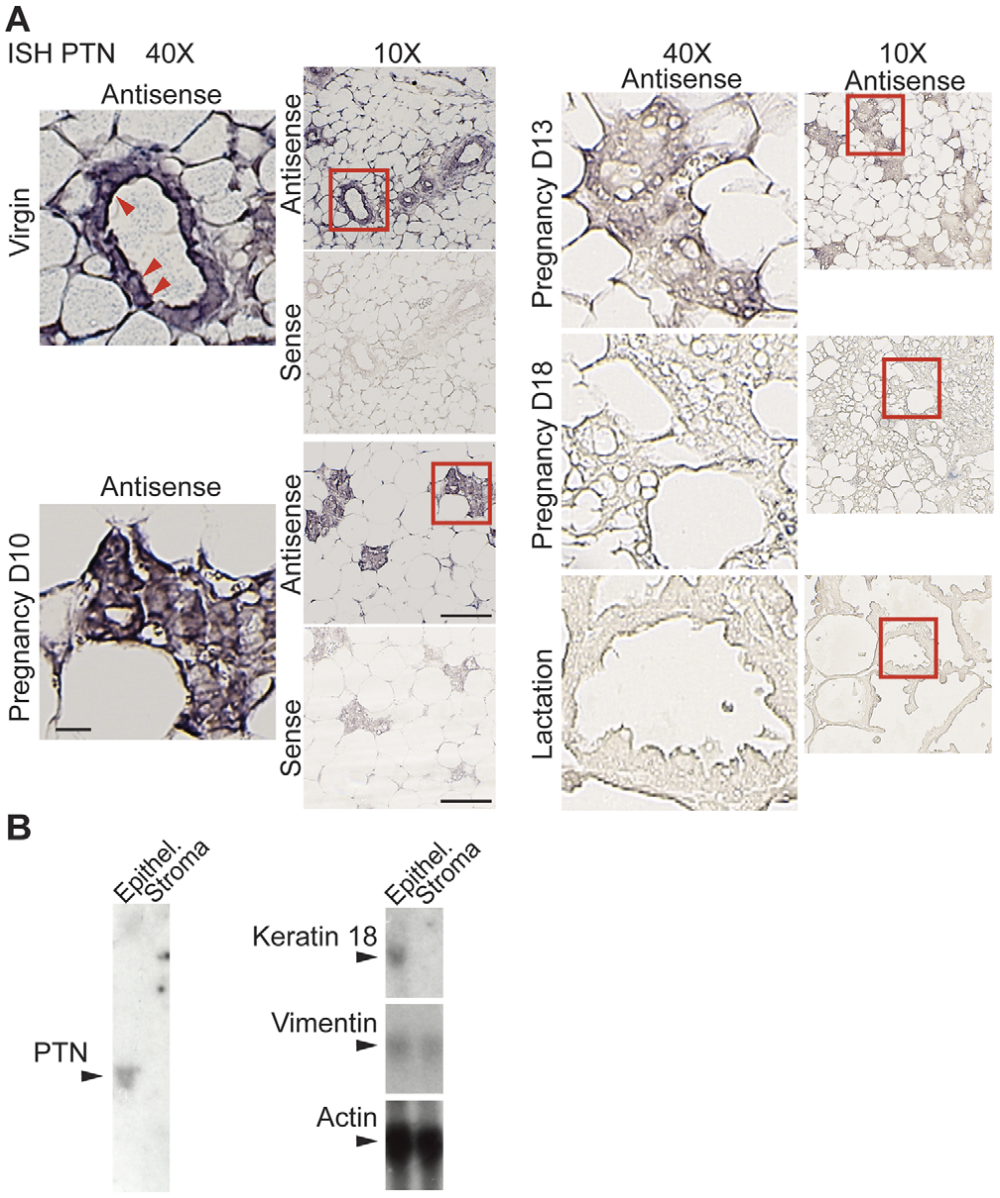
Mammary gland tissue distribution of PTN mRNA. (A) In situ hybridization (ISH) with PTN mRNA antisense and sense probes. Red squares indicate the areas shown at higher magnification. Scale Bars  = 25 μm and 100 μm respectively. Red arrowheads indicate PTN mRNA expression in mammary epithelial cells. (B) Northern blot for PTN mRNA in virgin mammary glands fractionated into epithelial and stromal cells.

PTN mRNA is highly regulated during pregnancy and reduced 30-fold by day 15 with the ALK receptor regulated in parallel reduced 100-fold by day 15 (Figure 1A,B). In agreement with a previous report [42], PTN and its receptor ALK mRNA levels were not affected during the first 10 days of gestation when the mammary gland is characterized by proliferating ductal epithelial cells. To determine which cells mostly express and secrete PTN, mRNA expression was analyzed by *in situ* hybridization as well as cell fractionation followed by Northern blot (Figure 2). *In situ* hybridization for PTN mRNA supports the downregulation during pregnancy and shows major expression in the epithelial compartment of the mammary gland (Figure 2A). Also, mammary glands from virgin mice were digested with collagenase to isolate epithelial cells from the glands. Northern blots showed Keratin 18 expression in the epithelial fraction and detectable expression of PTN only in this fraction. Actin and vimentin showed similar expression in both of the fractions (Figure 2B).

**Figure 3:**
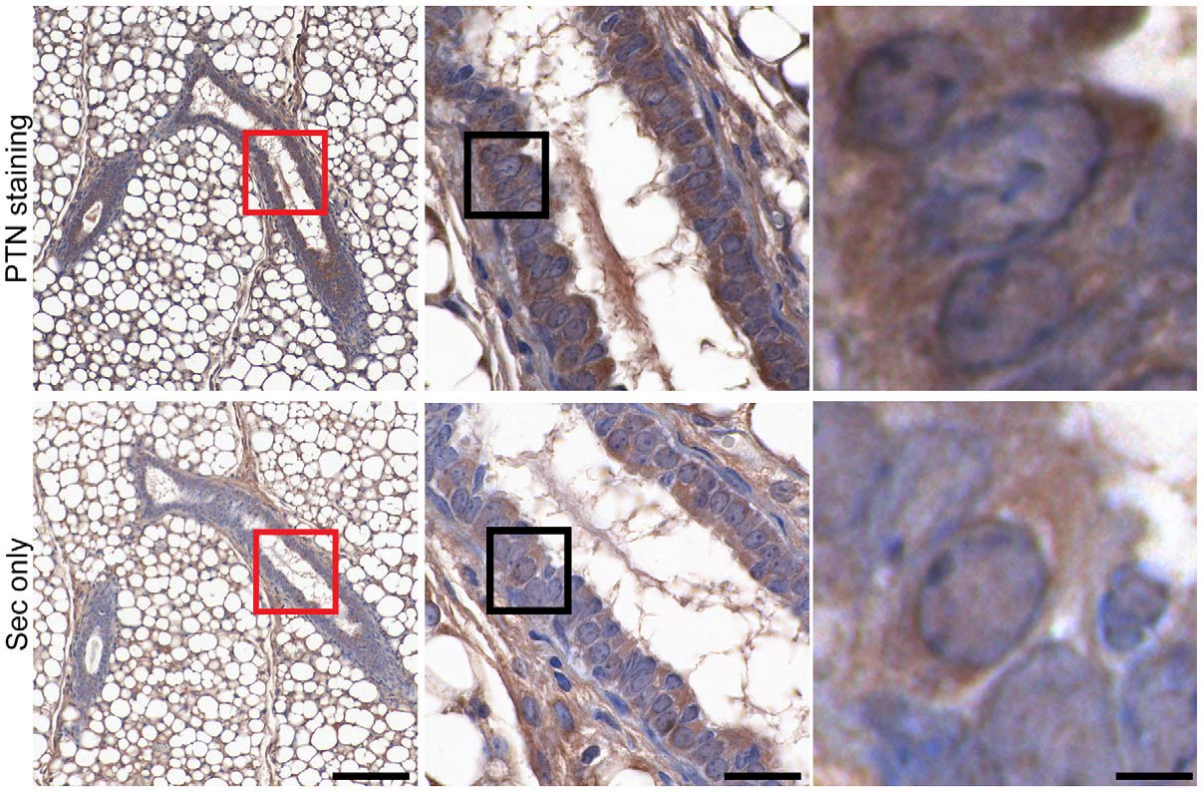
PTN protein expression in mammary glands and mammary epithelial cells (MECs). (A) Immunohistochemistry of virgin mouse mammary glands with a polyclonal anti-PTN antibody or secondary antibody only (sec. only) as a negative control. The dark brown staining shows PTN crossreactivity. Scale Bar  = 50 μm. Magnified ductal areas are indicated by the red squares (Scale Bar  = 25 μm) and individually magnified duct epithelial cells by the black squares (Scale Bar  = 10 μm).

**Figure 4:**
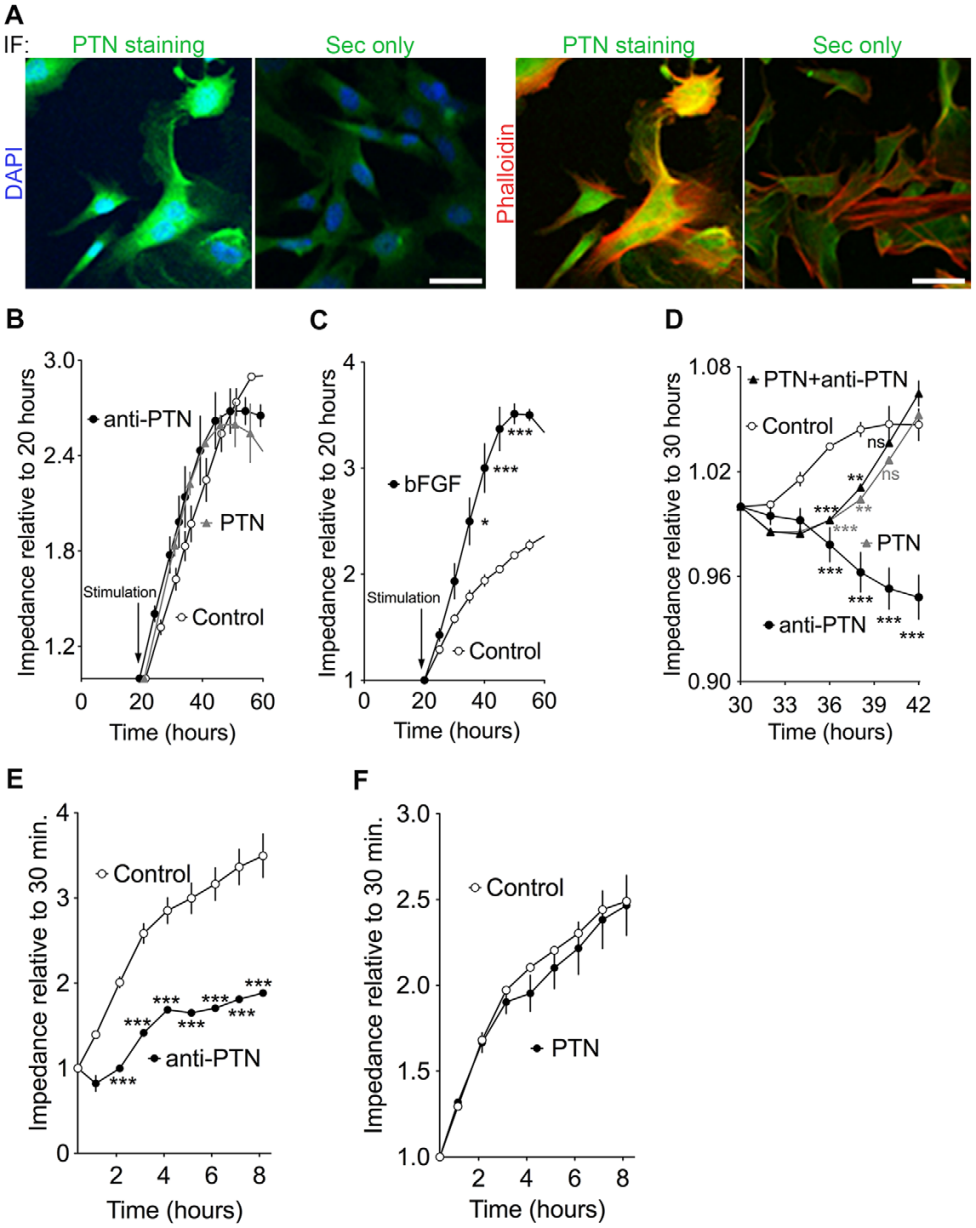
PTN protein expression and function in mammary epithelial cells (MECs). (A) Immunofluorescence (IF) staining of MECs grown on poly-D-lysine coated slides. PTN (green), DAPI (blue), Phalloidin (red). Images are shown as merged images of anti-PTN with DAPI and anti-PTN with Phalloidin staining. Scale Bar  = 20 μm. (B, C) Real-time impedance sensing of MECs growth relative to stimulation time (20 hours) with anti-PTN blocking antibody (anti-PTN), recombinant PTN (PTN), or antibody elution buffer (Control) (B), bFGF or resuspension buffer (C). (D) Real-time impedance sensing of MECs migration relative to 30 hours from plating and four hours from scratching. MECs were treated with anti-PTN blocking antibody (anti-PTN), recombinant PTN (PTN), alone or in combination or antibody elution buffer (Control). (E) Real-time impedance sensing of MECs invasion relative to 30 minutes from plating. MECs were treated with anti-PTN blocking antibody (anti-PTN), or antibody elution buffer (Control) (E), recombinant PTN (PTN) or PTN resuspension buffer (Control) (F). The data are representative of at least three independent experiments done in duplicates. ***P<0.001 versus control by ANOVA.

The PTN protein is released from cells and bound to heparin sulfate-containing proteoglycans in the extracellular matrix [32]. Immunohistochemistry and Immunofluorescence shows PTN protein staining in the mammary duct epithelium and in cultured primary mammary epithelial cells (MECs) (Figure 3 and 4A). In the mammary tissue staining, PTN protein is detected preferentially in duct epithelia though stromal tissues also show immunoreactivity very likely due to secreted PTN that is stored locally (Figure 3). In cultured MECs co-localization with DAPI and Phalloidin shows the PTN protein mostly in the extracellular matrix (Figure 4A). Together with the *in situ* hybridization for PTN mRNA, these data suggest that PTN is mostly expressed in the epithelial cells of the mammary gland, released from there and stored in the tissues.

Hormonal changes induced by breeding have been linked to changes in the mammary gland composition [48] but there was no statistically significant difference in PTN mRNA expression levels between mammary gland of virgin and pseudopregnant mice. Also, after the first pregnancy, PTN and ALK expression returned to pre-pregnancy levels (Figure 1). However, after repeated pregnancies, we saw a 8-fold downregulation of PTN mRNA in the mammary gland of exbreeders compared to virgins (mean ± SEM qPCR cycle: virgin, −4.96±0.34; exbreeders, −1.98±0.57; n = 4–5 age-matched mice per group; P = 0.004, t-test). Our findings are in agreement with D'Cruz et al. [42] who previously described permanent changes induced by parity in gene expression of growth factors such as PTN and suggested an increased differentiation state of the mammary gland after multiparity. We propose that the decrease in PTN expression in the mammary gland during mid-pregnancy and after repeated pregnancies is due to increased differentiation of mammary epithelial cells and a loss of undifferentiated epithelial cells (see below).

### PTN activity in primary mammary epithelial cells (MECs)

**Figure 5:**
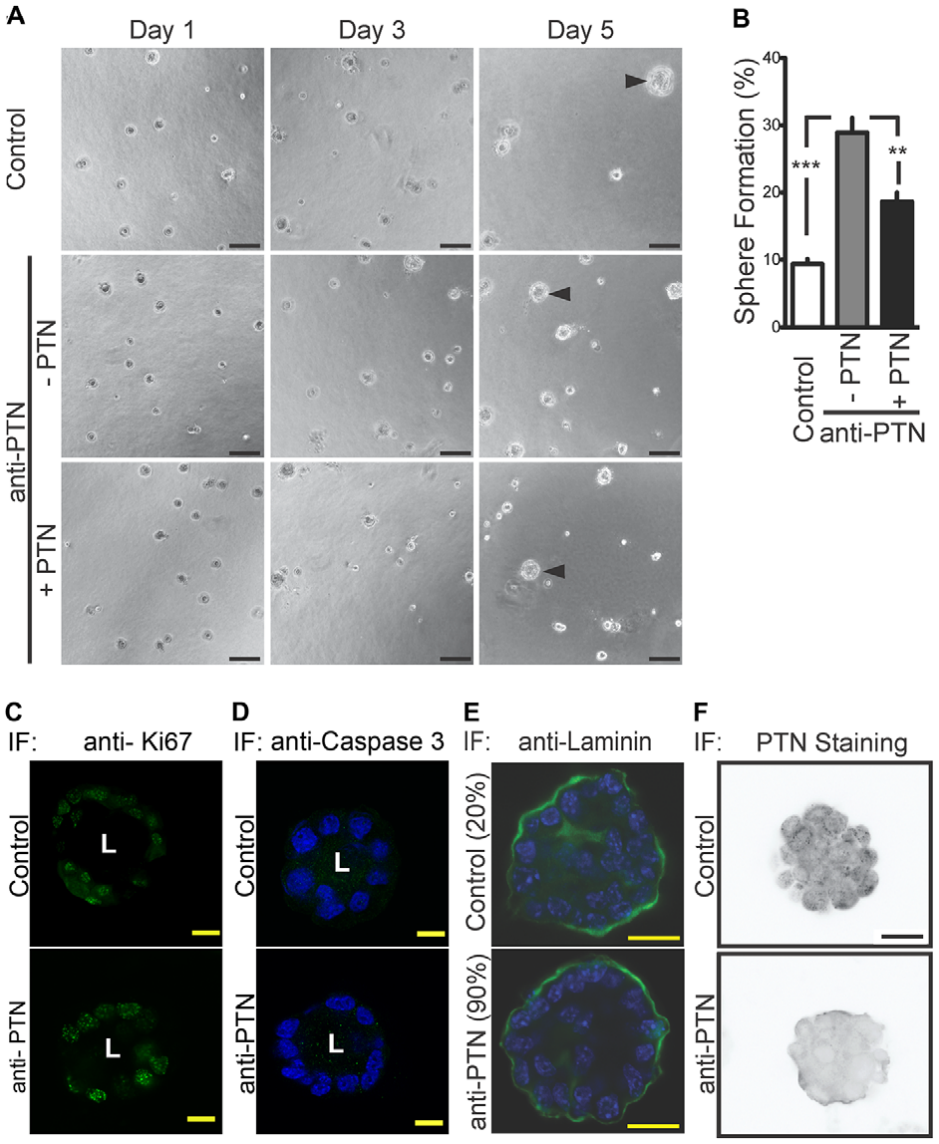
PTN effect on mammosphere formation. (A) Time course of mammosphere formation day 1, 3 and 5 without and with a blocking anti-PTN antibody ± recombinant PTN protein. Representative mammospheres that meet the threshold surface area of >900 µm^2^ are indicated by an arrowhead. Pictures are representative of 3 independent experiments. Scale bar  = 50 µm. (B) Quantification of mammospheres (>900 μm^2^ ) formed per field at day 5. Data are means ± SE; n = 5 fields/well; 4 wells for each condition. **P<0.01; versus anti-PTN treated cells; ***P<0.001 versus control by ANOVA. (C to F) Immunofluorescence of mammospheres at Day 5 for Ki67 (green)(C), Caspase 3 & DAPI (green & blue) (D), laminin & DAPI (green & blue) or for PTN (grey scale). Staining is shown in mammospheres treated with blocking anti-PTN antibody (anti-PTN) versus control. Scale bar = 20 µm.

Since PTN expression was detected mostly in the mammary epithelial cells, we studied its potential contribution using a PTN blocking antibody in cultured primary mammary epithelial cells (MECs; Figure 4,5). We first investigated whether PTN activity regulates MECs growth using the real-time monitoring electric impedance approach (Figure 4B,C). Treatment with PTN blocking antibody or stimulation with PTN recombinant protein did not change the MECs proliferation index (Figure 4B). The addition of bFGF, another growth factor, enhanced MECs proliferation (Figure 4C). This suggests that MECs growth was still responsive to growth factors stimulation. These findings were confirmed by Cell Titer Glow assay, another standard procedure to monitor cell proliferation (data not shown). As mentioned in the Introduction, PTN has been shown to be a promoter of invasion and metastasis in different tumor types including breast cancers [19], [22], [34], [35]. Real-time monitoring electric impedance shows that PTN activity regulates MECs migration and invasion (Figure 4D,E,F). Treatment with the anti-PTN antibody inhibited MECs migration (Figure 4D; p<0.001). After an initial inhibitory effect on MECs migration (p<0.001), the addition of recombinant PTN restored MECs migration to control levels after 12 hours and reversed the effect of the blocking antibody on MECs migration. These findings suggest that MECs motility is dependent on endogenous PTN activity. Invasion studies measured by real-time monitoring electric impedance show that PTN activity maintains MECs invasion. Overall these findings suggest a role for PTN as a promoter of MECs motility and invasion. Growing MECs in a three-dimensional mesh such as Matrigel has been shown to maintain the signaling cues for cellular organization and differentiation that are more representative of *in vivo* conditions than when grown on a plastic surface [49], [50], [51], [52]. MECs treated with the blocking antibody formed a significantly higher number of mammospheres per field (Figure 5A,B; p<0.001) compared to controls. Adding recombinant PTN protein to the MECs treated with the anti-PTN antibody reversed the effect of the antibody (Figure 5B; p<0.01). Also, immunofluorescence showed PTN staining in the control and not in the anti-PTN treated mammospheres as evidence of sufficient dosing of the anti-PTN antibody (Figure 5F). This confirms that mammosphere formation potential of MECs is controlled by endogenous PTN activity. Immunofluorescence staining for the proliferation marker Ki67 shows not apparent difference between control and anti-PTN treated spheres (Figure 5C). This concords with what we observed in the proliferation studies with MECs grown on plastic (Figure 4B). Immunofluorescence staining for the apoptosis marker Caspase 3 suggests that PTN does not regulate MECs apoptosis (Figure 5D). We observed that MECs were able to form mammospheres with a lumen surrounded by a monolayer of polarized cells and laminin secreted into the extracellular matrix irrespective of PTN activity (Figure 5E) [53]. However, whereas most (∼90%) of anti-PTN treated mammospheres showed detectable laminin deposition, this was observed in only a few (∼20%) of the control spheres. From this, we conclude that PTN activity in MECs disrupts cell adhesion to the extracellular matrix via reduced laminin deposition and thus results in fewer spheres with correct apical organization. We speculate that PTN activity inhibits the deposition of extra-cellular matrix components such as laminin and thus its function elicits cells migration and invasion of MECs grown on a plastic surface.

### PTN function during mammary gland development

In contrast to other organs, most mammary gland development happens after birth during puberty when the ovaries begin hormone production. In mice, the primordial mammary ductal tree starts proliferating, branching and invading the mammary fat pad after weaning at approximately three weeks of age when hormonal stimuli start. By 9 to 12 weeks, the epithelial ducts have fully invaded the fat pad and become quiescent until pregnancy occurs.

**Figure 6:**
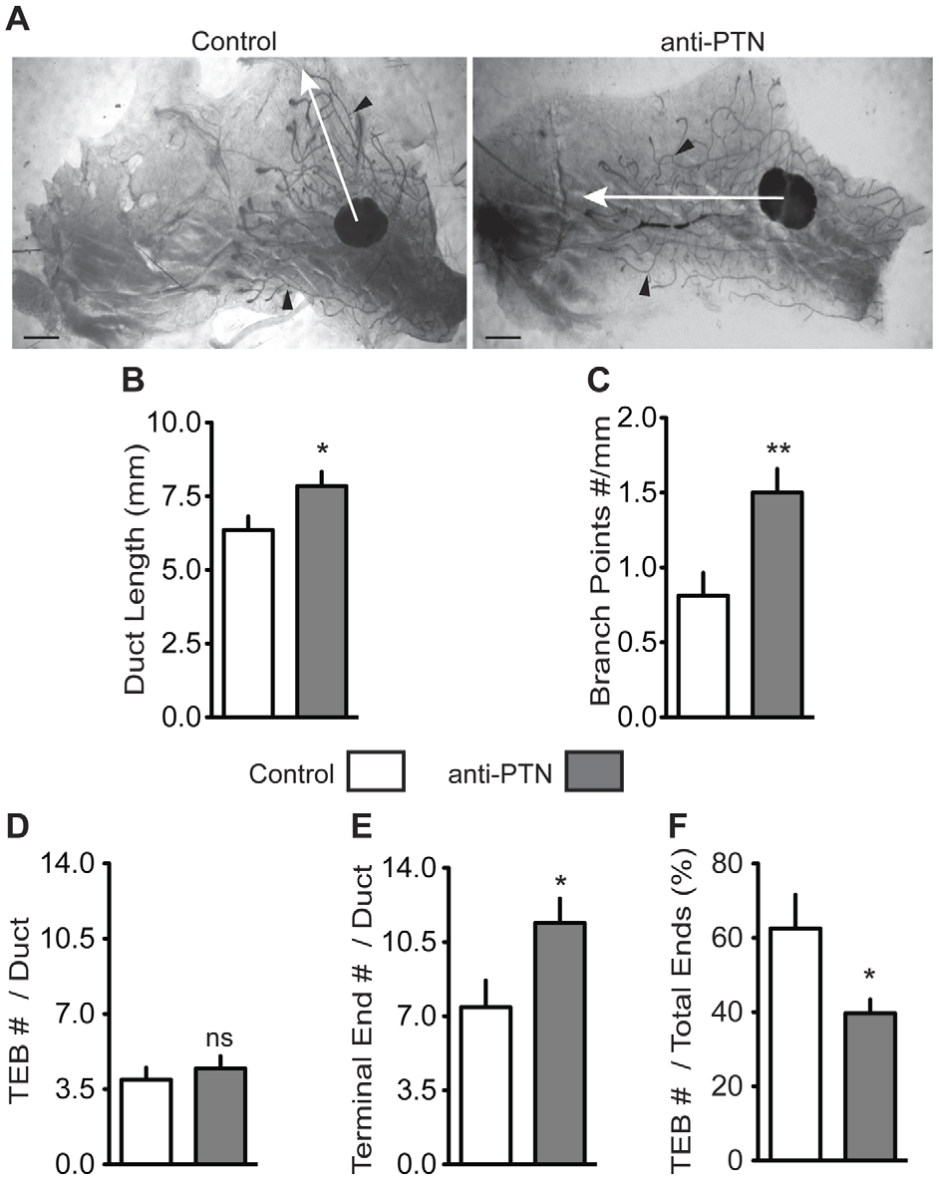
PTN effect on ductal outgrowth and branching in developing mammary glands. (A) Whole mounts of representative mammary glands from mice treated with anti-PTN blocking antibody versus control. White arrows: distance of the outgrowth of a representative duct measured from the midpoint of the lymph node. Black arrowheads: branch points. Scale bar  = 1.5 mm. (B to F) Controls (n = 9 mice, white), anti-PTN (n = 10 mice, grey). (B, C) Ductal length and number of branch points with the ductal length expressed in mm and the number of branch points per mm of duct. Data are means ± SE; *P = 0.04; ** P = 0.006; n = 3 ducts per mouse. (D to F) Quantitation of Terminal Ends (TE) and Terminal End Buds (TEBs) of mammary ducts; (D) TEBs per duct, ns =  not significant; (E) TEs per duct, * P = 0.03; (F) TEBs per total number of duct ends ( = TEBs + TEs), *P = 0.027. Data are means ± SE; n = 6 ducts per mouse.

Treatment of mice with the PTN blocking antibody introduced in the *in* vitro studies above was used to investigate PTN function during mammary gland development. The antibody K_D_ is 1 nM and we detected serum concentrations of 1 to 10 µM in the treated mice one week after the last dose (Figure S1B). This suggests that sufficient dosing was used to block endogenous PTN. Mice treated during the crucial phase of mammary duct invasion into the fat pad from 3 to 6 weeks of age showed an increase in ductal penetration of the mammary fat pad and in the average number of branch points per duct (Figure 6A to C). In a further analysis we assessed additional well-defined functional units in the developing mammary gland, Terminal End Buds (TEBs). TEBs are highly proliferative club-shaped structures containing stem cells [54], [55], [56] and drive the penetration of the mammary ducts into the fat pad. Their number is high during early puberty [54] and morph into alveolar and terminal buds as the gland matures [54], [57]. Mice treated with the anti-PTN antibody showed an increase in the number of Terminal Ends but not in TEBs (Figure 6D,E). The percentage of TEBs has been suggested as an indicator of the developmental stage of the mammary gland [55]. Indeed, control mice showed a 1.8-fold higher fraction of TEBs suggesting that PTN activity inhibits the differentiation of TEBs into Terminal Ends (Figure 6F). H&E staining confirmed that both treated and control groups had indistinguishable, regular mammary duct structure (data not shown). In mice treated with anti-PTN for an additional 3 weeks until 9 weeks of age no differences in the final ductal and branching structures of the mammary glands were observed between treatment and controls (data not shown). From these data we conclude that PTN delays ductal outgrowth, branching and terminal end formation without impacting the final gland development.

### PTN signaling pathways in the developing mammary gland

**Figure 7:**
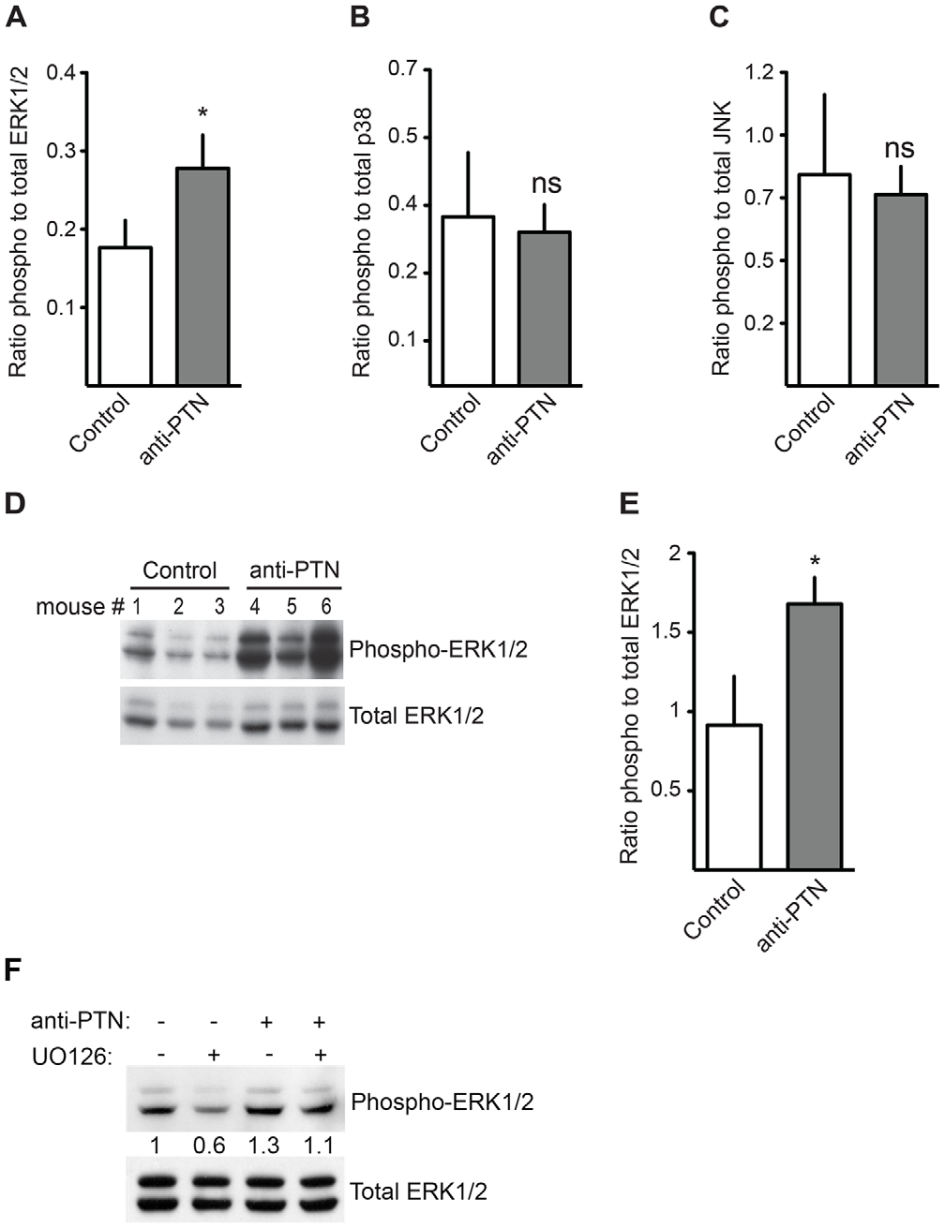
PTN effect on MAP kinase pathway activity in developing mammary glands. Phospho-ERK1/2 (A), phospho-p38 (B), and phospho-JNK (C) in whole mammary gland extracts from anti-PTN treated mice (n = 10) versus controls (n = 9). The ratio of phospho- to total protein was obtained from multiplex assays. Ns, not significant; *P<0.05. (D, E) Western blot analysis for pERK1/2. Representative Western blots (mouse m1 to m6) and quantitation of phospho ERK1/2 relative to total ERK1/2 in 3 independent experiments (n = 9 to 10 per group). (F) Representative Western blots of whole cell lysates from MECs treated for 24 hours with either UO126 (500 nM) alone or in combination with anti-PTN blocking antibody and quantitation of phospho ERK1/2 relative to total ERK1/2. Numbers represent the ratio of the quantification of phospho ERK1/2 to total ERK1/2. Data are means ± SE; *P<0.05.

The MAP kinase pathway is used by hormones and growth factors that control mammary gland development (for a review see [58]). Since the treatment with the PTN blocking antibody showed that PTN delays ductal outgrowth, branching and terminal end formation during pubertal development, we investigated if the enhanced maturation of the glands observed with this treatment was accompanied by a change in pathway activation. The analysis of whole mammary gland lysates of anti-PTN treated versus control mice showed an increase in phospho-ERK1/2 but not in the phospho-p38 and phospho-JNK pathway (Figure 7). This suggests that PTN inhibits ERK1/2 signaling in the mammary gland during ductal outgrowth and branching but does not impact JNK and p38 MAP kinases. We investigated if PTN inhibits ERK1/2 signaling in MECs. Western blot analysis of whole cell lysates of MECs treated with anti-PTN, the ERK1/2 inhibitor UO126, alone or in combination showed that blocking PTN activity increases phospho-ERK1/2 signaling compared to control and that reverses the inhibitory effect of UO126 to baseline (Figure 7F).

We conclude that PTN inhibits ERK1/2 signaling in the mammary epithelial compartment of the mammary gland during ductal outgrowth and branching.

### PTN activity prevents mammary epithelial cell differentiation

**Figure 8:**
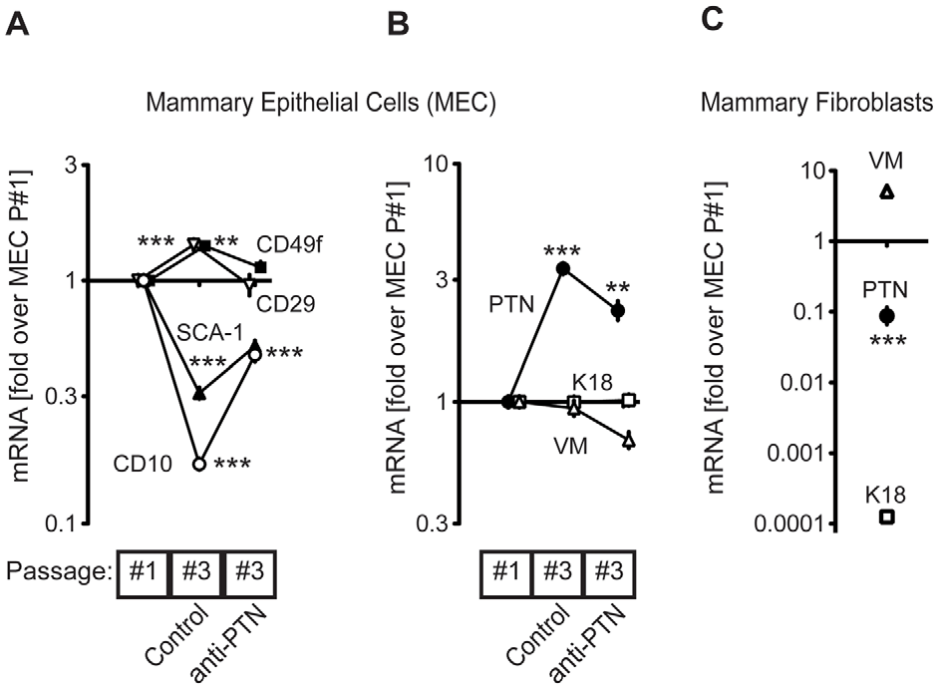
PTN effect on cultured mammary epithelial cell (MEC) differentiation. (A, B) Effect of treatment of MECs with a blocking anti-PTN antibody. (A) Progenitor marker mRNA, (B) Keratin 18 (K18), Vimentin (VM) and PTN mRNA expression were measured by quantitative, real-time PCR. The effect of continuous passaging of MECs ± anti-PTN is shown relative to passage #1 MECs. Data are means ± SE; n = 4 and 2 independent experiments for VM and K18 respectively; **P <0.01; ***P <0.001 by ANOVA. MECs passage#1 expression relative to Actin: CD29, 3.5; CD49f, 5.6; CD10, 6.6; SCA-1, 2.3; K18, 2.9; VM, 5.3; PTN, 12.2. Control MECs passage#3 expression relative to Actin: CD29, 2.9; CD49f, 5.1; CD10, 9; SCA-1, 3.8; K18, 2.9; VM, 5.5; PTN, 10.3. Treated MECs passage#3 expression relative to Actin: CD29, 3.5; CD49f, 5.4; CD10, 7.5; SCA-1, 3.2; K18, 2.9; VM, 5.9; PTN, 10.9. (C) Expression of K18, VM and PTN in mammary fibroblasts relative to MECs passage #1. Expression of K18, VM and PTN in mammary fibroblasts relative to Actin: PTN, 15.7; K18, 15.9; VM, 3.

As shown above, blocking endogenous PTN activity caused an increase in mammosphere formation and in maturation of the mammary gland during postpuberal development. To further understand the role of PTN in this process, we monitored the impact of anti-PTN treatment on the expression of genes indicative of the progenitor cell status using cultured MECs (CD29, CD49f, SCA-1 and CD10; Figure 8). The respective markers are described in Refs. [59], [60], [61], [62], [63], [64], [65], [66] and reviewed further below. There was no significant difference in the mRNA expression levels of the luminal epithelial marker K18 or the mesenchymal marker vimentin when comparing the effect of anti-PTN treated MECs with MECs at passage #1 (Figure 8B). Also, vimentin expression in mammary fibroblasts was ∼10-fold higher than in the MECs at passage #1 and we can thus exclude the possibility of a significant fibroblasts contamination of the MEC preparation (Figure 8C).

High CD29 and CD49f and low SCA-1 have previously been associated with the presence of mammary stem cells (MaSC) [61], [62]. In addition, CD29 and CD49f belong to the integrin family and their activity is important for the attachment of cells to the extracellular matrix [67]. CD10, a known myoepithelial marker, was recently suggested as a progenitor cell marker with peptidase activity and has been shown to have a role in cleaving signaling molecules that induce differentiation [62], [66]. The expression of CD29 and CD49f slightly increased while that of SCA-1 and CD10 decreased during the passaging and the 3D culturing of control MECs from passage #1 to #3 (Figure 8A). However, MECs treated with the PTN blocking antibody, showed no significant changes (CD29, CD49f) or only small changes (SCA-1, CD10) relative to MECs at passage #1 (Figure 8A). As mentioned above, treated MECs were able to form a higher number of spheres with correct apical organization and laminin deposition compared to control MECs (Figure 5). Overall our data suggests that endogenous PTN expression maintains the epithelial cells in an undifferentiated phenotype and that blocking its activity stimulates the expansion and differentiation of mammary progenitor cells as indicated by the altered CD29, CD49f, SCA-1 and CD10 expression profile. A role for PTN as a stem and progenitor cell regulator has already been described in the brain, bone and bone marrow [29], [31], [68] our data also suggest such a role for MECs.

## Discussion

PTN is expressed and highly regulated during embryonic and postnatal development of different tissues [13]. During adulthood, PTN expression is confined to the nervous system, the iris, the uterus and the basal layer of the tongue epithelium [13], though several reports showed PTN expression also in the mouse mammary gland and in the human breast [39], [40], [41]. The detection of PTN expression in primary breast cancers and in estrogen receptor negative breast cancer cell lines and its pro-angiogenic and pro-metastatic function [19], [22], [34], [35] make PTN a good candidate for therapeutic targeting. Here we sought to understand PTN function in the normal mammary gland.

The mammary gland is one of the few organs that mature after birth and continues development during puberty under hormonal stimuli until adulthood when it becomes quiescent. During pregnancy, the mammary gland goes through several changes such as proliferation, differentiation and apoptosis of the alveolar cells. The multiple cell populations in the mammary gland change in number and ratio during the process of development as well as during pregnancy. Using different, independent approaches we show here that PTN is predominately expressed in epithelial cells in the virgin mammary gland. The tissue analysis was also validated in isolated mammary epithelial cells (MECs) harvested in parallel with isolated fibroblasts showing a >10 fold higher expression of PTN in the MECs than in fibroblasts (Figure 8C) and expression of PTN protein at the cell membrane (Figure 4A).

PTN expression remained unaltered during the first 10 days of pregnancy but was downregulated 30-fold during late pregnancy when the mammary gland is proliferating and differentiating in preparation for lactation. A downregulation of PTN expression during late pregnancy was surprising to us because the epithelial ducts are still proliferating and PTN has previously been shown to be a growth factor for different types of cells [19]. We surmise that PTN expression is sustained by the first wave of proliferation of the epithelial cells during early pregnancy when the cells are still in an undifferentiated state but downregulated during the second wave of proliferation in late pregnancy when the epithelial cells are undergoing lobular-alveolar differentiation.

There is only one known PTN homologue, midkine (MK), that shares 50% protein identity [69], has a similar activity profile [1] but an approximately 10-fold lower affinity for the ALK receptor when compared with PTN [70], [71]. In Northern blots MK was below detection in virgin, midgestation, or lactating mammary glands (data not shown) although data mining of published cDNA array studies suggests a regulation of MK in the mammary gland during pregnancy (NCBI GEO GDS2843). We conclude from our functional data and a comparison with MK that PTN is the predominant ligand of this growth factor family in the mammary gland.

PTN was found to be a ligand that binds to the extracellular domain of the tyrosine kinase receptor ALK [72]. Others have described that the protein tyrosine phosphatase receptor ζ1 (PTPRζ1) is involved in the control of the PTN/ALK signaling [73]. Marzan et al. showed expression of PTPRζ1 in HC11, a mammary epithelial cell line, and suggested a role for the interaction of PTN and PTPRζ1 in the migration of the mammary epithelial cells [40]. Here we describe a striking parallel regulation of PTN and ALK expression in the mammary gland. This suggests to us that ALK constitutes a major signaling pathway utilized by PTN during mammary gland development.

When comparing PTN expression among nulliparous, uniparous, multiparous and pseudopregnant mice, a significant decrease in PTN mRNA expression level was detected in the mammary gland of multiparous versus nulliparous mice but not in pseudopregnant mice. These findings suggest a regulation of PTN expression due to gestational hormones and a decrease in PTN expression possibly due to a loss in mammary epithelial progenitor cells by repeated pregnancies. Full term pregnancy has been demonstrated as a protective factor against the risk of breast cancer [47]. Studies in rodent mammary glands and in the human breast suggest that the differentiation status induced during repeat pregnancies contributes to this protective effect. We suggest that permanent changes in the expression of local regulatory factors such as PTN, may be important in the progression to this protected state. In fact, at least one other study has implicated the downregulation of a variety of growth-promoting factors including PTN, amphiregulin and IGF-1 due to parity as a cause for the protective effects [42]. Furthermore, we have seen that PTN is overexpressed in approximately 60% of human breast carcinoma [19], [74] and in chemically-induced rodent models where parity was seen to have a protective effect against tumorigenesis [19]. Thus, it is tempting to speculate that the downregulation of PTN, an anti-differentiation factor, has a critical role in this protective effect.

PTN mRNA expression in the mammary epithelial cells has been previously suggested [41] but its function in these cells was unclear. Treatment with the anti-PTN blocking antibody in vivo indicates that PTN inhibits the expansion of the epithelial ductal tree and the activation of the ERK1/2 signaling pathway during pubertal development of the mammary gland. Three-dimensional cultures of MECs treated with the PTN blocking antibody and expression analysis of markers associated with progenitor mammary epithelial cells such as CD29, CD49f, SCA-1 and CD10 indicate that PTN activity maintains the mammary epithelial cells in an undifferentiated state. A model for the cooperation between CD10 and CD29 in maintaining the homeostasis of mammary epithelial stem cells by inhibiting differentiation has been proposed where CD29 is responsible for keeping the stem cells anchored to the extra-cellular matrix and CD10 for creating a gradient of cleaved protein with differentiation activity [65]. The mRNA expression profile in the progenitor cell markers suggests that the anti-PTN antibody treated MECs have a more differentiated phenotype than the control cells and are more similar to the MEC at passage #1.

The fact that the anti-PTN antibody treated MECs formed a higher number of mammospheres with correct apical polarity and laminin basement membrane deposition than control MECs in 3D cultures suggests that PTN inhibits polarization of MECs. PTN expression has been shown in neuronal progenitor cells and its activity enhances progenitor cells expansion and differentiation in different tissues [29], [31], [68]. A recent study has shown that PTN deficiency in the stroma both maintains the hematopoietic stem cell (HSCs) population during serial transplantation and increases their repopulating activity by promoting cell proliferation and differentiation and altering gene and protein expression of stem and progenitors cells [75]. That PTN could have opposing effects depending on dose and expression of different receptors has been previously proposed [75]. Treatment with the anti-PTN blocking antibody of mammary epithelial cells grown on a plastic surface showed that endogenous PTN sustains cell motility and invasion (Figure 4B to F). This finding seems to be in contradiction with the in vivo studies where treatment with the anti-PTN blocking antibody indicates that PTN inhibits the expansion of the epithelial ductal tree during pubertal development of the mammary gland (Figure 6). Overall our findings suggest that PTN has opposing effects depending on the cell surroundings. Blocking PTN increases MECs sphere potential by stimulating better cell-matrix adhesion in MECs grown in 3D. We propose that PTN disrupts MECs adhesion to a plastic surface resulting in sustained cell migration and invasion while in vivo this effect is overcome by the interaction with the surrounding stroma. Our study shows that PTN is a regulator of progenitor cells in the mammary gland and that its expression maintains the mammary epithelial cells in an undifferentiated state while blocking its activity causes an expansion and differentiation of mammary epithelial cells. We proposed that the increased in maturation of the mammary gland and in mammosphere formation of the anti-PTN antibody treated MECs is due to an expansion of progenitor epithelial cells that are farther committed in the epithelial lineage that have appropriate epithelial polarization and cell attachment to the ECM, a more differentiated phenotype and higher expression of the protease CD10. Asselin-Labat et al. [76] suggest that the paracrine expression of factors from the luminal epithelial hormonal responsive cells is important in keeping the homeostasis of the mammary epithelial progenitor cells [76]. We suggest that PTN is one of these regulatory factors of progenitor cells that are expressed by the mammary epithelial cells under hormonal stimuli.

### Conclusion

PTN appears to inhibit differentiation of mammary epithelial cells and maintain their progenitor phenotype that is evidenced during mammary gland development as well as in isolated mammary epithelial cells in culture. It is tempting to speculate that PTN downregulation observed with multiparity is protective towards breast cancer, possibly by reducing the immature precursor population susceptible to malignant transformation and by expanding differentiated epithelial cells.

## Materials and Methods

### Ethics Statement

All procedures using animals were approved by the Georgetown University IACUC and were conducted according to the NIH guidelines for the care and use of laboratory animals.

### Mice

C57BL/6, Balb/c and FVB mice were purchased from the Jackson Laboratory. All procedures using animals were approved by the Georgetown University IACUC and were conducted according to the NIH guidelines for the care and use of laboratory animals.

### RNA detection

Mammary glands or brains were isolated from female balb/c mice. Involution days are given relative to the weaning of 21 day old pups with mammary glands from exbreeders harvested >4 weeks after weaning. All experiments were performed using combined abdominal and thoracic mammary glands. Tissues were homogenized in RNA-STAT, and the RNA was isolated via a standard chloroform extraction procedure. For experiments requiring tissue fractionation, mammary glands were finely minced at 4°C and then incubated in DMEM Hams F12 media (Gibco, BRL) supplemented with 0.15% collagenase (type A, Boehringer Mannheim) for 1 hour at 37°C. After collagenase digestion, glands were passed through cheesecloth (Baxter) and filtered through a cell strainer using a 70 μm mesh (Falcon).

The filtrate was centrifuged for 5 minutes at 500×g (stromal cell fraction). Both the filtrate pellet and the filtered collected fraction (epithelial organoids) were resuspended in RNA-STAT and processed for RNA extraction as described above.

Northern blots were performed as previously described [19]. RNA blots of 5 μg total RNA/lane were hybridized with P^32^ labeled probes primed from full length cDNA for mouse Keratin 18 [77] or mouse vimentin [78] or human beta actin (Clontech). In Situ Hybridization (ISH) for PTN mRNA was done with digoxigenin labeled riboprobes as previously described [79]. For quantitative real-time PCR (qRT-PCR) RNA from thoracic mammary glands or from mammospheres was used. Mammospheres were harvested from three-dimensional cultures and Matrigel was removed as described elsewhere [80]. Reverse transcription (RT) of 1 µg of total RNA was performed using iScript^TM^ gene expression was quantified using SYBR green (BioRad). Primers were:

PTN (Forward) TGGAGAATGGCAGTGGAGTGTGT

PTN (Reverse) TGGTACTTGCACTCAGCTCCAAACT

K18 (Forward) CAAGTCTGCCGAAATCAGGGACG

K18 (Reverse) TCCAAGTTGATGTTCTGGTTTTTCATGG

ALK (Forward) GGACGGGACACAGCTCCATG

ALK (Reverse) GCACTCCAGACCATATCGACTGCG

CD10 (Forward) CTCTCTGTGCTTGTCTTGCTC

CD10 (Reverse) GACGTTGCGTTTCAACCAGC

CD29 (Forward) ATGCCAAATCTTGCGGAGAAT

CD29 (Reverse) TTTGCTGCGATTGGTGACATT

CD49f (Forward) TGCAGAGGGCGAACAGAAC

CD49f (Reverse) GCACACGTCACCACTTTGC

SCA-1 (Forward) AGGAGGCAGCAGTTATTGTGG

SCA-1 (Reverse) CGTTGACCTTAGTACCCAGGA

Primers for Vimentin were described in [81].

### Immunohistochemistry

Abdominal mammary glands were fixed in formaldehyde and embedded in paraffin. Immunohistochemical analysis of 5 μm section was done using the Vectastain ABC Elite System (Vector Labs). The primary goat anti-human PTN antibody (R&D) diluted 1∶500 was incubated at 4°C overnight. The slides were counterstained with hematoxylin (Sigma Aldrich). Tissue sections are representative of 3–4 mice. Image capturing for stained section was performed with a Nikon E600 Epifluorescence Microscope.

### Whole Mount

Mammary gland #4 was excised from 6-week-old mice whole mount stained [82] and ductal penetration and branching points were quantified as described in [83]. Ductal penetration was expressed as the average length (mm) of the three longest epithelial ducts in each mammary gland. Branch points of the measured ducts were counted and divided by the average duct length (mm) in each mammary gland. Terminal End Buds (TEBs) were defined as having a largest diameter ≥100 µm [54]. Terminal Ends (TEs) were defined as having a largest diameter <100 µm. This included terminal ducts and alveolar buds. TEB and TE numbers are given per duct. The percentage of TEBs was calculated relative to the total number of ductal ends, i.e. TEBs plus TEs [55].

### Anti-PTN Antibody Production, Administration and Detection

The hybridoma cell line producing the anti-PTN mouse monoclonal antibody (clone 3B10) was previously described [84] and was cultured in DMEM (Invitrogen) media supplemented with 10% fetal bovine serum in a CELLine Device (BD Biosciences). Media containing the antibody was collected every 7 days, pooled and purified using a 5 ml HiTrap Protein G HP Column (GE Healthcare). Antibody concentration was assessed against a bovine serum albumin standard after SDS-PAGE and Coomassie staining. Antibody binding to PTN was titrated using an ELISA with immobilized PTN and yielded a K_D_-value of 1 nM.

FVB mice (3 weeks old) were injected intraperitoneally with a total dose of 2 mg/kg of the antibody or with PBS as a control. Serum samples from control and treated 6-week-old FVB mice were diluted 1∶100 in PBS and tested in an ELISA assay with immobilized recombinant human Pleiotrophin (PTN; at 25 ng/well; R&D Maxisorb Nunc plates). 5% (w/v) non-fat dry milk was used to block non-specific binding. Superfluous solution was washed off with 3 washes with 0.05% Tween20 (Fisher Scientific) in PBS ( = PBST). Diluted serum samples were incubated for 1 h at room temperature. Anti-PTN antibody stock solution diluted to 100 nM was used for calibration. Wells were washed 5 times with PBST. A horseradish peroxidase-linked sheep, anti-mouse IgG was the secondary antibody (Amersham Biosciences, GE Healthcare), 1-Step Turbo TMB ELISA Substrate (Thermo Scientific) the detection reagent and sulfuric acid the stop solution. The absorbance was read at 450 nm.

### Western Blot and Phosphorylation Studies

Whole mammary glands #3 from 6-week-old FVB mice was harvested and lysates (5 µg/well) were analyzed either using a MS6000 MAP Kinase Whole Cell Lysate kit (Phospho protein) and MS6000 MAP Kinase (Total Protein) Whole Cell Lysate kit (Meso Scale Discovery) according to manufacturer's instructions. Whole mammary gland lysates and whole cell lysates from primary cultures were run (75 µg/lane) on a 4–20% Tris-Glycine NOVEX gel (Invitrogen), transferred to PVDF membranes. Primary epithelial cells were harvested 24 hours after treatment with UO126 (500 nM) alone or in combination with anti-PTN blocking antibody. Immunoblot studies for phospho-MAPKs and total MAPKs were performed with the respective rabbit polyclonal antibodies (1∶1000 dilution; Cell Signaling). Visualization was performed using Immobilon Western HRP substrate Peroxide Solution (Millipore) with horseradish peroxidase-linked sheep anti-rabbit immunoglobulin G as a secondary antibody (Amersham Biosciences, GE Healthcare).

### Primary Mammary Epithelial and Fibroblast Cell Harvest and Culture

Mammary glands #3 and #4 were dissected from 7 to 9-week-old FVB mice carefully removing muscles, lymph nodes and nipples. Glands were minced and digested in 1 mg/ml collagenase (Sigma-Aldrich) solution for 90 minutes at 37°C on a shaker at 150 rpm. The digested tissue was centrifuged for 10 minutes at 600×g and washed 4 times. DMEM/F12 (Invitrogen) media supplemented with Penicillin/Streptomicin (Invitrogen) 1X and 50 µg/ml Gentamicin (Invitrogen) was used to prepare the collagenase solution and to wash the digested tissues. Cells were resuspended and plated on collagen-coated plates (Costar) in MEC growth media (Ham's F12 (Invitrogen) supplemented media with 10% fetal bovine serum, 16 µg/ml insulin (Invitrogen), 10 ng/ml EGF (Roche Applied Biosciences), 1 µg/ml hydrocortisone (Sigma-Aldrich), 50 µg/ml gentamicin (Invitrogen), 4 ng/ml cholera toxin (Sigma-Aldrich) and 1X penicillin/streptomycin). To avoid fibroblast contamination, cells in the supernatant were transferred to uncoated plates 20 minutes after initial plating. Fibroblasts growing on collagen coated plates (BD Biosciences) and epithelial cells were incubated at 37°C and 5%CO_2_ and cultured up to passage #3 and then used for RNA extraction, immunofluorescence, three-dimensional culture, real-time impedance sensing and Western blot analysis.

### Cell Proliferation, Migration and Invasion Assay: Electric Impedance Sensing

Primary epithelial cells were plated on microelectrodes embedded wells and the impedance of the cell monolayer was monitored in real time as described elsewhere [85], [86]. For proliferation and migration assay, 8-10,000 cells per well were plated in a xCelligence E-culture plate array (Roche Applied Biosciences) and followed until confluence and steady state impedance. For proliferation assay, cells were stimulated 20 hours after plating with either blocking PTN antibody alone or in combination with recombinant PTN protein (R&D) or Glycine/Tris-HCl pH 7.5 antibody elution buffer or bFGF (Invitrogen) and cell monolayer impedance was monitored until confluence. For migration assay, cell monolayers were allowed to form and were disrupted by scratching with a sterile pipette tip as described elsewhere [87]. For invasion assay, 100,000 cells per well were plated in a xCelligence CIM-culture plate array. Blocking PTN antibody, recombinant PTN protein (R&D) and Glycine/Tris-HCl pH 7.5 antibody elution buffer (Control) were used as chemo attractant/repellent. Impedance was measured for the first 6 hours of cell invasion with xCelligence Instrument (Roche).

### Mammosphere Three-dimensional (3D) Culture

3D culture was performed as described in [50]. Briefly, cells were resuspended in HAM's F12 supplemented media containing EGF at 20 ng/ml, 1.25% FBS, 2% Matrigel (BD Bioscience) and either blocking PTN antibody alone or in combination with recombinant PTN protein (R&D) or Glycine/Tris-HCl pH 7.5 antibody elution buffer (Control). 400 µl of the cells/media mix was added per well in an 8 well Nunc Lab-Tek II Chamber Slide System precoated with Matrigel. Cells were seeded at 8,000 cells/well. Media containing either the antibody or the antibody with equimolar recombinant PTN protein or the elution buffer was replenished every two days. Pictures of the wells were taken at day 1, 3 and 5.

### Immunofluorescence

Primary mammary epithelial cells passage #3 grown in poly-D-lysine coated 8 well Lab-Tek II Chamber Slide System (Nunc) were fixed and permeabilized twice with 4% formaldehyde, 0.2% Triton X in cold PBS for 10 minutes at room temperature, washed with ice-cold PBS, blocked with 1% BSA in PBS and incubated with a goat anti-human PTN (1∶100; R&D) antibody for 1h at room temperature. After three washings with PBS, cells were incubated with Alexa Fluor 488 anti -goat IgG (1∶1000; Invitrogen) and with Alexa Fluor 568 Phalloidin (1∶50; Invitrogen) for 1h at room temperature. After three washings with PBS, cover slips were mounted with ProLong Gold Antifade with DAPI (Invitrogen) and fluorescence was analyzed on an Olympus Fluoview FV300 Laser Confocal Microscope (Olympus, Hamburg, Germany).

Immunostaining of mammospheres was performed as described in [80]. Briefly, Matrigel was dissolved with PBS-EDTA and mammosphere suspensions were transferred to poly-D-lysine coated slides. Mammospheres were fixed in 4% formaldehyde for 20 minutes at room temperature. Fixative solution was then aspirated and 100 µl/sample of 50 mM NH_4_Cl was added to samples for 5 minutes. Samples were washed 3 times with PBS and then immunostained as described in [80]. Spheres were stained using as primary antibody ki67, Caspase 3 mouse anti-human laminin γ2 (D4B5 clone; 1∶200; Millipore) and goat anti-human PTN (1∶100; R&D) diluted in blocking buffer and incubated with Alexa Fluor 488 anti-mouse IgG (H+L) (1∶1000; Invitrogen). Cover slips were mounted as above.

### Statistical and Image Analysis

Prism 5 for Mac (GraphPad, San Diego, CA) was used for all statistical analyses. Adobe Photoshop CS3 was used to process the pictures for immunohistochemistry, whole mounts, Western blot, mammosphere three-dimensional culture and immunofluorescence. Brightness/Contrasts and Levels adjustments were applied to the whole image. All images were adjusted to the same level. Image J was used to measure the ductal length, terminal end buds and terminal ends diameter, and mammosphere surface area. Immunofluorescence images were overlaid using the Metamorph Software.

This article contains supporting information.

## Supporting Information

Figure S1 **Anti-PTN antibody binding activity to PTN and its detection from mouse serum of treated mice.** (A) Anti-PTN antibody binding to immobilized PTN by ELISA. Anti-PTN antibody K_D_-value  = 1 nM. Control =  antibody elution buffer. OD defines absorbance at 450 nm. (B) PTN blocking antibody detection from mouse serum of treated mice (anti-PTN; n = 9 mice, grey) versus control (n = 5 mice, white) by PTN immobilized ELISA. Absorbance at 450 nm is defined as arb.u. Data are means ± SE; ***P = 0.0004; two-tailed student-t test.
(TIF)
