# Correction: Pleiotrophin (PTN) Expression and Function in the Mouse Mammary Gland and Mammary Epithelial Cells 

**DOI:** 10.1371/annotation/55d244a2-0960-4f5f-95e9-2eea136654b1

**Published:** 2013-02-08

**Authors:** Sonia M. Rosenfield, Emma T. Bowden, Shani Cohen-Missner, Krissa A. Gibby, Virginie Ory, Ralf T. Henke, Anna T. Riegel, Anton Wellstein

There was an error in the title of the article. The correct title is: Pleiotrophin (PTN) Expression and Function in the Mouse Mammary Gland and Mammary Epithelial Cells 

