# Supplementary figures and images for: Pleiotrophin (PTN) Expression and Function and in the Mouse Mammary Gland and Mammary Epithelial Cells
Source: PLoS One. 2012 Oct 15;7(10):e47876. doi: 10.1371/journal.pone.0047876 (PMC3471873; doi:10.1371/journal.pone.0047876)

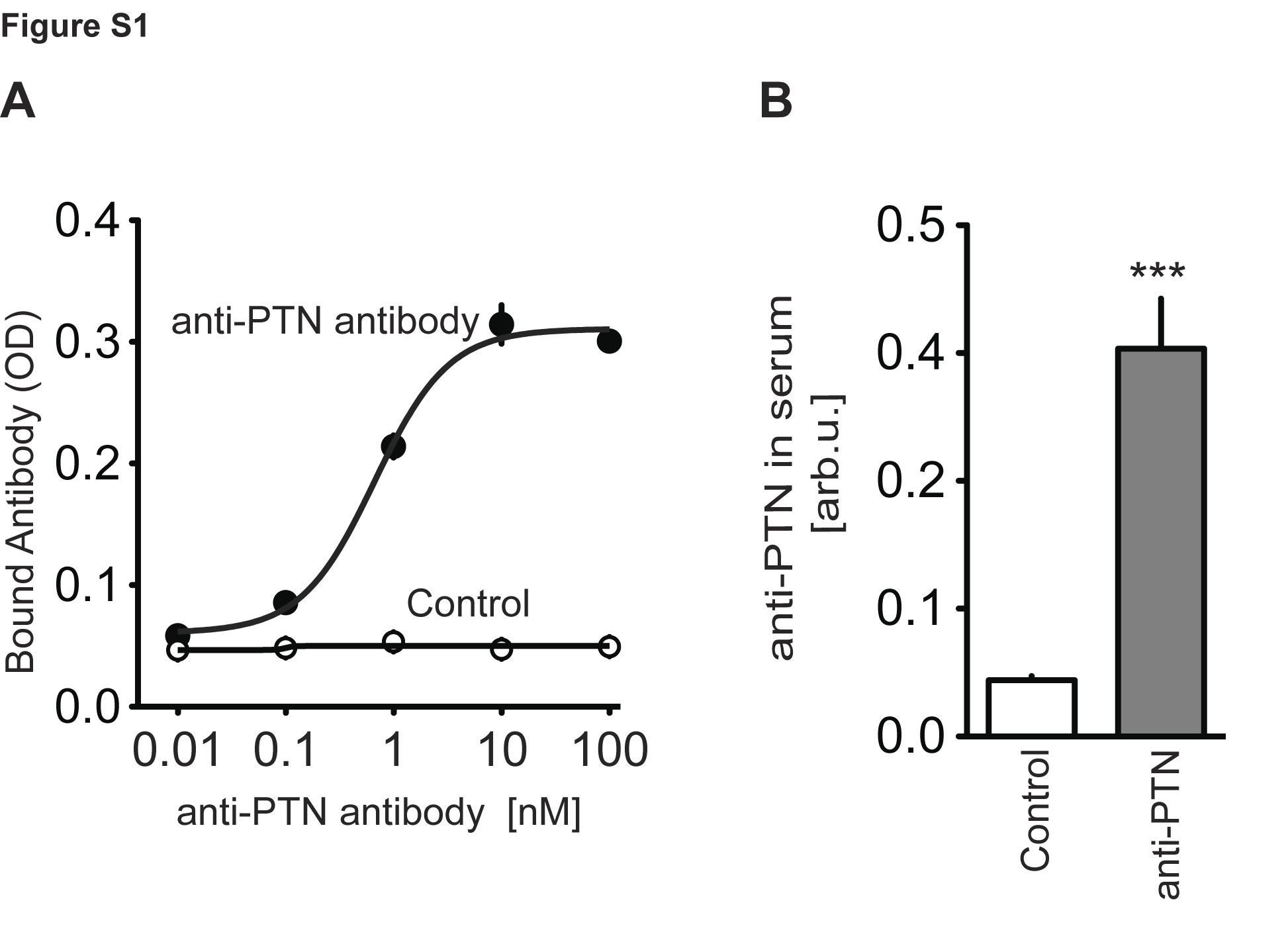

Supplement: Figure S1 — Anti-PTN antibody binding activity to PTN and its detection from mouse serum of treated mice. (A) Anti-PTN antibody binding to immobilized PTN by ELISA. Anti-PTN antibody KD-value = 1 nM. Control = antibody elution buffer. OD defines absorbance at 450 nm. (B) PTN blocking antibody detection from mouse serum of treated mice (anti-PTN; n = 9 mice, grey) versus control (n = 5 mice, white) by PTN immobilized ELISA. Absorbance at 450 nm is defined as arb.u. Data are means ± SE; ***P = 0.0004; two-tailed student-t test. (TIF) [file pone.0047876.s001.tif]
